# Maternal color‐consciousness is related to more positive and less negative attitudes toward ethnic‐racial outgroups in children in White Dutch families

**DOI:** 10.1111/cdev.13784

**Published:** 2022-05-11

**Authors:** Judi Mesman, Ymke de Bruijn, Daudi van Veen, Fadime Pektas, Rosanneke A. G. Emmen

**Affiliations:** ^1^ 4496 Leiden University College Leiden University The Hague The Netherlands

## Abstract

A prerequisite to anti‐racist socialization in families is acknowledging ethnic‐racial (power) differences, also known as color‐consciousness. In a sample of 138 White Dutch families from the urban Western region of the Netherlands with children aged 6–10 years (53% girls), observations and questionnaires on maternal color‐consciousness and measures of children's attitudes toward Black and Middle‐Eastern ethnic‐racial outgroups were collected in 2018–2019. Variable‐centered analyses showed that maternal color‐conscious socialization practices were related to less negative child outgroup attitudes only. Person‐centered analysis revealed a cluster of families with higher maternal color‐consciousness and less prejudiced child attitudes, and a cluster with the opposite pattern. The mixed results emphasize the importance of multiple methods and approaches in advancing scholarship on anti‐racism in the family context.

The concept of anti‐racism reflects the insight that not being racist is insufficient, because the failure to actively and explicitly address dominant structures and discourses surrounding ethnic‐racial inequalities only reinforces the (racist) status quo (Bonilla‐Silva, 2015; Kendi, 2019; Lewis et al., 2020; Neville et al., 2013). An important first step in anti‐racism is acknowledging that racial categories were socially constructed to justify the oppression and exploitation of other humans for profit (e.g., Kendi, 2019; Lewis et al., 2020; Richeson & Sommers, 2016). Research shows that White majority parents predominantly support an ideology in which ethnic‐racial difference should not be seen or talked about, claiming that they do not “see” color, and therefore treat everybody equally (e.g., Katz, 2003; Vittrup, 2018). However, given the overwhelming evidence of systemic racism in Western societies (e.g., Feagin & Elias, 2013; Lentin, 2008), silence about ethnic‐racial difference ignores—and, therefore, perpetuates—very real differences in the societal experiences of people with different ethnic‐racial identities and can arguably be defined as racist in and of itself (Bonilla‐Silva, 2015; Richeson & Sommers, 2016). Thus, anti‐racist socialization of children in the family context can only be achieved when parents first acknowledge ethnic‐racial difference.

## Key concepts

The current study aims to examine color‐conscious (vs. color‐evasive and power‐evasive) attitudes and practices of White Dutch mothers in relation to their 6‐ to 10‐year‐old children's attitudes toward ethnic‐racial outgroups. The terminology regarding ethnic‐racial ideologies varies between authors, and is evolving. In the current paper, we use the term color evasion to denote what Neville et al. (2013) describe as silence about ethnic‐racial differences, emphasizing sameness, and often representing an attempt to reduce prejudice and/or a way to avoid the potential discomfort that talking about race and ethnicity can elicit. We also follow Neville et al. (2013) in using the term power evasion to refer to the denial of racism and prejudice that contributes to and reinforces power differences between ethnic‐racial groups. We use the umbrella term color‐consciousness to reflect ideologies that acknowledge ethnic‐racial differences and/or power imbalances between ethnic‐racial groups in society (e.g., Bell, 2016; Richeson & Nussbaum, 2004; Vittrup, 2018). It must be noted that color‐consciousness does not equate anti‐racism (Berman & Paradies, 2010), but is rather a prerequisite for anti‐racism. Similarly, children's positive or negative attitudes about ethnic‐racial outgroups that are examined in this study are not synonymous with (anti‐)racist attitudes or behaviors, but represent the building blocks for processes of (a lack of) ethnic‐racial othering that become salient when encountering peers from ethnic‐racial outgroups.

Although we follow the literature in using ‘color’ to refer to evasive versus conscious approaches to issues of race, we use the adjective ethnic‐racial in relation to ideologies, attitudes, and socialization to reflect the fact that racism is not only about color but also about other ethnic dimensions that characterize marginalized groups in White‐dominant societies (e.g., Meer, 2013). Cultural and religious characteristics of certain groups (e.g., Muslims) may be more salient markers of their outgroup status in the eyes of the ethnic‐racial majority than color in certain context, in particular the European one, and are subject to essentialist racialization that justifies the term racism to refer to their marginalization (Meer, 2013). In addition, the word “race” (“ras” in Dutch) is rarely used in the Netherlands because of its associations with the atrocities of the Holocaust and colonial times. We, therefore, opt to combine the word ‘racial’ commonly used in the international scientific iterature with the term “ethnic”’ that is more commonly used in everyday Dutch language, resulting in “ethnic‐racial” as the adjective to describe key concepts and variables.

## Color‐consciousness and its effects

Turning to research on the prevalence of ethnic‐racial ideologies, it appears that color evasion is the norm among White people in the United States, that it is found using a variety of measures, and as early as in late childhood—early adolescence (Apfelbaum et al., 2008; Neville et al., 2013; Norton et al., 2006). Importantly, consistent with the anti‐racist framework, there is evidence that a color‐conscious ideology has more positive effects on intergroup relations and (reduced) prejudice than color‐evasive ideologies in adults (Whitley & Webster, 2019). In addition, there is evidence that a color‐evasive approach to ethnic‐racial diversity is not conducive to positive experiences for underrepresented racial groups in various experimental and real‐life settings (e.g., Apfelbaum et al., 2008; Holoien & Shelton, 2012; Jansen et al., 2016; Norton et al., 2006). Given these patterns, being raised in a context of color evasion may also engender more negative attitudes about ethnic‐racial outgroups in children.

In general, children's attitudes and ideologies are indeed influenced not only by key socialization agents such as parents (Graham et al., 2020) but also by teachers and peers (e.g., Lee et al., 2012). However, before adolescence, parents play a more central role in children's lives than others do, as they are the ones who decide on the school that a child attends, and at least partly influence friend formation through their own chosen social environment and selective facilitation of contact (Ladd et al., 2016). In addition, the childhood period before adolescence is particularly relevant for studying intergroup attitudes, given meta‐analytic evidence that ethnic‐racial prejudice develops early and peaks between 5 and 7 years, with a slight decrease between 8 and 10 years, and no developmental changes in adolescence (Raabe & Beelmann, 2011). Furthermore, parental and child intergroup attitudes are already significantly related in early childhood as shown by a meta‐analysis (Degner & Dalege, 2013). The link between family socialization context and children's ethnic‐racial attitudes has also been convincingly shown in research by Hagerman (2014), who found clear alignment of children's views on race and ethnic‐racial parenting strategies. Thus, a focus on parents and their primary school children can offer insights into the beginnings of ethnic‐racial attitude development, and the role of socialization patterns in that period.

A growing literature on children's exposure to ethnic‐racial socialization also highlights the predominance of the color‐evasive ideology among parents (Bell, 2016; Katz, 2003; Loyd & Gaither, 2018; Vittrup, 2018). White parents who employ color‐conscious socialization have been found to be more aware of their own potential ethnic‐racial biases (Perry et al., 2019), more conscious about racism in society (Hagerman, 2014), and on average also show less biased racial attitudes (Zucker & Patterson, 2018). In an intervention study among White families in the United States by Vittrup and Holden (2011), children whose parents’ diary‐reported discussing race‐related themes with them in response to video materials showed more positive outgroup attitudes than those whose parents did not. However, there is evidence that color evasion is so ingrained in White majority families that ceiling effects (i.e., almost all parents showing high color evasion) hamper proper analyses (Pahlke et al., 2012; Vittrup & Holden, 2011). In a more recent study, American White children's anti‐Black attitudes significantly decreased after they had a conversation with their parents about racial bias, guided by a specially designed method to facilitate such conversations (Perry et al., 2020). The studies discussed so far were all conducted in the United States, and studies in Europe are rare.

The assessment of parental color‐consciousness (or color evasion) varies considerably across studies, but most focus on explicit measures reflecting self‐reported or observed ideologies regarding the topics of race and racism (e.g., Pahlke et al., 2012; Perry et al., 2019; Vittrup, 2018; Zucker & Patterson, 2018). Given evidence that implicit rather than explicit parental attitudes may be more predictive of children's attitudes (e.g., Pirchio et al., 2018), exploring different parental measures in relation to children's attitudes can contribute to our understanding of ethnic‐racial socialization mechanisms. Several studies in the domain of parenting and child behavior problems suggest that effects of parenting are stronger when parental behaviors match parental beliefs (e.g., Barnett et al., 2010). Children might be most positively affected when one type of color‐conscious socialization co‐occurs with one or more other types. Thus, the interactions between various measures of parental attitudes and practices (or their additive occurrence) may predict child attitudes above and beyond their potential separate effects.

## The Dutch context

Many European countries, such as the Netherlands, are racially diverse and experience interracial tension (Sociaal Cultureel Planbureau, 2020), but few studies have examined ethnic‐racial socialization in that context. The European context in general and the Dutch context in particular is both similar and different compared with the North‐American one in various ways when it comes to ethnic‐racial issues. Similarities lie in the presence of systemic racism in Europe (Essed & Hoving, 2014; Ghorashi, 2014; Wekker, 2016) as for example documented in empirical studies of ethnic‐racial discrimination in the labor market (e.g., Thijssen et al., 2021) and the housing market (e.g., Aalbers, 2007). Another similarity is the fact that underrepresented ethnic‐racial groups have a lower average socioeconomic status than the White majority and are more likely to live in poverty in Europe in general (Phalet et al., 2015) and the Netherlands in particular (Centraal Bureau voor de Statistiek, 2020). Differences lie in the historical background and nature of ethnic‐racial diversity, as well as in the public discourse about ethnic‐racial discrimination.

The ethnic‐racial majority in the Netherlands is the White Dutch group. From that perspective, the main ethnic outgroups in terms of numbers are the Afro‐Dutch group and the Middle Eastern group. The Afro‐Dutch group consists of Surinamese‐Dutch and Caribbean‐Dutch populations (mostly due to postcolonial migration), and a variety of African groups, such as people from Somalia, Cape Verde, Ghana, and Angola (economic migrants and refugees). The Middle Eastern group consists mainly not only of the Turkish and Moroccan populations (mostly due to labor migration) but also of people from Syria, Afghanistan, and Iraq (mostly refugees). The Middle‐Eastern group predominantly identifies as Muslim, a religious affiliation that applies to approximately 5%–6% of the Dutch population according to recent national surveys (Centraal bureau voor de Statistiek, 2020). Scholars have suggested that in Europe, the othering of ethnic‐racial outgroups is rooted particularly in perceptions of cultural difference, and a lack of shared identity or heritage of people with a migration background (Gobel et al., 2018).

Anti‐Black racism toward the Afro‐Dutch group is entangled with the history of colonialism and slavery in Surinam and the formerly Dutch part of the Caribbean region which has long been denied as having significance for the present (Essed & Hoving, 2014; Weiner, 2014). Although there have been voices calling for acknowledgment of and apologies for the atrocities of slavery for many decades, it is only very recently—and much later than in the United States—that these issues are high on the societal agenda through the work of movements such as Black Lives Matter and other emancipatory organizations.

The group with Middle Eastern roots in the Netherlands experiences discrimination that appears to be mostly about their religion and culture. As early as in the 1970s, there were protests against Turkish labor migrants, followed by an explicitly negative public and political discourse about Islam and Middle‐Eastern culture that have often been described as fundamentally incompatible with Dutch culture (Lucassen & Lucassen, 2015). A person's religion is not necessarily clear based on appearance alone, but research has shown that a Middle‐Eastern appearance or name lead to assumptions about (Islamic) religious affiliation, which in turn is subject to essentializing prejudice, and discrimination against this group (e.g., De Koning, 2020).

Around half of Afro‐Dutch and Middle‐Eastern Dutch people report experiencing discrimination (Sociaal Cultureel Planbureau, 2020). There is also a growing openly racist discourse in Dutch society that does not seem to be recognized or acknowledged as being racist (e.g., Ghorashi, 2014). Color evasion appears to be decreasing in public discourse in the Netherlands due to both anti‐racist movements and populist politicians with a racist discourse. To what extent color evasive or color‐conscious discourses in Dutch society are present in parent–child conversations is unknown, and of course likely to vary between families.

When it comes to White Dutch children's attitudes toward ethnic‐racial outgroups, several studies have revealed patterns of racial in‐group preference and outgroup rejection similar to those in the United States. White Dutch children and adolescents show ethnic‐racial in‐group favoritism, as well as ethnic‐racial outgroup rejection (De Bruijn et al., 2020; Fortuin et al., 2014; Gonzalez et al., 2008; Verkuyten, 2007; Verkuyten & Thijs, 2001). Whether these patterns are related to family socialization practices regarding race and racism is unknown, and no studies have examined whether ethnic majority Dutch parents talk to their children about these topics, or whether color evasion is the main strategy as found in most American studies. There is a study from Germany that found (indirect) effects of parental attitudes and parenting styles on the levels of White children's ethnic‐racial prejudice, mostly for fathers (Jugert et al., 2016). However, this study examined only children's ingroup preference, and not outgroup rejection, and focused on the East Asian outgroup whose position in European societies is historically quite different from the position of Black and Middle‐Eastern populations.

## The current study

In this study among White Dutch families, we examined observed and self‐reported maternal color‐conscious practices, (i.e., concrete behaviors related to the explicit acknowledgment of ethnic‐racial difference), as well as self‐reported color‐conscious attitudes (beliefs) focused on the acknowledgment of ethnic‐racial power differences. Although attitudes and behaviors are in theory linked (attitudes informing behavior), in practice the association between the two is generally weak in the realm of prejudice (e.g., Pearson et al., 2009), suggesting that they tap into different aspects of human functioning that need to be distinguished.

We tested the following hypotheses: (1) maternal color‐conscious practices and attitudes are associated with more positive attitudes about ethnic‐racial outgroups in their children; (2) maternal color‐conscious practices and attitudes are associated with less negative attitudes about ethnic‐racial outgroups in their children; (3) higher maternal color‐conscious practices and attitudes measured in different ways (observed and self‐reported) interact in predicting more positive and less negative outgroup attitudes in children (i.e., the predictive effect of one can be amplified by the presence of another); (4) higher maternal color‐conscious practices and attitudes, and more positive and less negative child attitudes toward ethnic‐racial outgroups cluster within individual families. We used both a variable‐centered (H1–H3) and a person‐centered approach (H4) to provide a comprehensive understanding of how the family characteristics under investigation might relate to one another assuming the sample reflects one population versus assuming the sample might reflect separate subpopulations (Henry et al., 2005).

## METHOD

### Participants

Families were recruited through child‐related events, organizations, social media, the researchers’ networks and snowball sampling. Inclusion criteria were: (1) the child was between 6 and 10 years old, (2) parents were the biological parent of the participating child and (3) living with the child, (4) parents did not have severe mental or physical illnesses, (5) the child did not have severe developmental disorders such as autism, and (6) families lived in the urban Western region of the Netherlands; (7) the parents, their parents and grandparents (i.e., the child's (great)grandparents, were born in a North‐Western European country).

The sample consisted of 147 families of children aged 6–10 years old. Because fathers participated in only about half of the families, we chose to focus our coding and analyses on mothers only at this stage of the project. Complete data for the current study variables were available for 138 mother‐child dyads. There were missing data for nine cases, of which four were due to missing Picture Book observations (see Measures), and five due to missing questionnaires. Because the Picture Book observations constitute our main predictor and one that has never been used before, we chose not to use imputation strategies for this variable. The five cases with missing questionnaires had not filled in any of the relevant questionnaires in the study, which severely limited sophisticated imputation strategies.

Countries of birth outside of the Netherlands of the (grand)parents included England, Belgium, Germany, Switzerland, Sweden, Spain, Norway, and Austria (eight mothers, seven fathers, ten maternal grandmothers, eight maternal grandfathers, five paternal grandmothers, six paternal grandfathers). Almost all parents self‐identified as culturally Dutch, suggesting long‐term residence in the Netherlands (and potentially only being born in a different country because of temporary residence of their families there). Five mothers and three fathers identified as either Belgian, German, or English (analyses with and without these cases revealed no changes in results).

Children's average age was 7.4 years (*SD* = 0.9) and included 54% girls. Mothers were between 26 and 48 years old (*M* = 39.9, *SD* = 4.3). Most mothers had a high level of education (bachelor's degree/higher vocational education or higher; 83%), and the family income was above the national average for 88% of the families. About a third of mothers identified as religious (30%). About half of the children (48%) attended schools with 25% or more of pupils of non‐Western background (a grouping label that is commonly used in categorizing Dutch schools, and broadly refers to pupils with a migration background from the Global South). We did not collect information about sexual orientation of gender identities.

### Procedure

During recruitment and the informed‐consent stage, families were told that they would participate in a study on the perceptions of parents and children on the multicultural society. Data were collected from May 2018 to February 2019. Families were visited at home by two researchers/research assistants. After obtaining consent from parents, several standardized parent‐child interaction tasks and child tasks were videotaped. In addition, parent(s) and child performed several computer tasks, and the parent(s) answered some questionnaires. The children completed the tasks under supervision of one of the home visitors. The parent was either in the same room in a different area, or in another room to complete her computer tasks or questionnaires. The week after the visit, parents were asked to fill out an online questionnaire. At the end of the visit, the child received a small gift (i.e., a toy or game worth ca 3 Euros). Parents received a gift card after filling out the online questionnaire. The study's procedures and methods were approved by the Ethics evaluation committee of the authors’ host institute that assesses the adherence to APA ethical standards in research with human subjects.

### Measures

#### Mothers’ color‐conscious practices—Observed

Mothers and children were videotaped while looking through a picture book that was specifically designed for the current study to elicit race‐related talk. The method was inspired by the work of Margie et al. (2005), and adapted to fit the Dutch ethnic‐racial context in terms of the largest ethnically underrepresented groups that have also been shown to be at the bottom of the ethnic hierarchy, namely the Afro‐Dutch (Antillean‐Dutch and the Surinamese‐Dutch) and the Middle Eastern Dutch (Turkish‐Dutch and Moroccan‐Dutch; Verkuyten & Kinket, 2000).

The book contains 10 pictures featuring two White, two Black, and two Middle Eastern children (a boy and a girl each, the Middle Eastern girl with a headscarf) in various situations. The first introductory picture features all six children standing next to each other. The following six pictures feature each of the six children alone in ambiguous situations (e.g., a mishap such as a broken vase that could reflect intentionally naughty behavior or an accident). The final three pictures show all six children and a male and female adult for each ethnic group in an explicitly cultural/religious setting relevant to the three represented ethnic groups (e.g., a Mosque, a Caribbean Carnival). The book did not contain any text. Mothers were instructed as follows: “Please go through each of the pictures and tell your child what you see. Your child may also want to talk about the pictures, which is fine of course, but we would like you to take the lead and tell a story about the pictures.” Example pictures can be found on the following website: www.judimesman.nl/opgroeien‐in‐kleur/.

All videotaped statements by mothers were first transcribed verbatim, and then coded. For all pictures, the absence/presence of statements about the ethnic appearance of the characters (three categories: skin color, hair/eye color or hair texture, head scarf), and the ethnic background of the characters (three categories: cultural heritage, religion, nationality) were coded. Multiple statements about the same category regarding the same character within one picture were only coded once. Example statements are as follows: “Two with a really dark skin, two white children, and two children from Morocco or Turkey I think”; “I see brown curls, and I see a headscarf”; “Look, they're all different, this one is Dutch, and this one might be from Africa”; “They are Muslims who invited people to dinner.”

Our research team developed a reliability set of 24 videos with consensus scores derived from the development phase in which the team piloted the coding system, established intercoder reliability within the team, and generated consensus scores through discussion in case of discrepancies. They then trained three (under)graduate students who also coded the reliability set. Intercoder reliability for all variables was adequate, with kappa values >.80. The trained coders then proceeded to code the videos for the rest of the sample. For the current analyses, we computed three total scores (for maternal color‐conscious practices by summing all variables reflecting absence/presence of statements about the ethnic appearance or background of the characters: one reflecting statements specifically about Black characters, one reflecting statements specifically about Middle‐Eastern children, and one reflecting statements about either or both total outgroup).

#### Mothers’ color‐conscious practices—Self‐reported

Mothers filled in the Parental Racial‐Ethnic Socialization Behaviors Questionnaire (Hughes & Johnson, 2001). For color‐conscious practices in White families, four items were selected that reflect talking to children about important people or events of different ethnic groups, and talking about discrimination against another ethnic group, or treating people from different ethnic groups equally. These were the following items: (1) Talked with your child about important people or historical events of various different ethnic groups; (2) Read books with your child about other ethnic groups; (4) Talked with your child about discrimination against another ethnic group; (7) Did or said something to show that everyone is equal, regardless of their cultural background. Because of the age of the children in this sample, we rephrased the items regarding encouraging children to read books to reflect reading books together. All items were scored on a 5‐point scale ranging from never to more than seven times. The internal consistency of the 4‐item scale was good, with Cronbach's *α* = .78.

#### Mothers’ color‐conscious attitudes

After the home visit, mothers also filled in 12 items from the Color‐Blind Racial Attitudes Scale (Neville et al., 2000) sent to them digitally, a questionnaire tapping into presence/absence of power‐evasive attitudes, that consists of items asking about racism in society and can be reversed to reflect color conscious attitudes. Items were selected based on reported factor loadings above .60 (Neville et al., 2000), one item was excluded because of limited applicability to the Dutch context (i.e., on the role of race in the type of social services people receive), leaving 11 items, and 1 item was adapted (‘Dutch should be the only *spoken* language’, rather than official language). The items are scored on a 1–5 Likert scale ranging from 1—strongly disagree to 5—strongly agree. Example items are: White people in the Netherlands have certain advantages because of the color of their skin; Social policies, such as affirmative action, discriminate unfairly against White people (reverse item). A total score was computed by summing the items (after reversing where necessary). The internal consistency of the total scale was satisfactory, with Cronbach's *α* = .78.

#### Children's interethnic attitudes: Outgroup preference and rejection

Children completed a social preference task based on the work by Levy et al. (2005) with 12 pictures: two boys and two girls of three ethnic groups (White, Black, and Middle Eastern). The children in the pictures all wore white t‐shirts, faced the camera straight, smiled and were placed against white backgrounds. The 12 pictures were presented together, and children were asked several questions representing preference and rejection: Who would you (not) like to sit next to in class? (instruction to select one or none), Who would you (not) like to invite for a play date at your house? (instruction to select one or none), and Who would you want to invite for your birthday party? (instruction to choose any number of children or none). Outgroup preference scores reflect the frequency of selecting a Black or Middle Eastern child to sit next to, play with, or invite to a birthday (separate per outgroup and combined, potential score range 0–10). Outgroup rejection scores (separately for Black and Middle Eastern outgroups, and combined) reflect the frequency of selecting an ethnic‐racial outgroup child to not sit next to or to not play with (potential score range 0–2).

#### Children's interethnic attitudes: Positive and negative outgroup attitudes

Children also completed a version of The Multiple‐Response Racial Attitude (Aboud, 2003) measure. This is an attribution task in which they were asked to assign five positive descriptors (e.g., nice, friendly) and five negative descriptors (e.g., naughty, mean) to any number of six children on photographs. These pictured children represented the same groups as in the social preference task (one boy and one girl from the White, Black, and Middle Eastern groups). Participating children could also choose to not assign a descriptor to any of the children and could instead assign the descriptor to the ‘rubbish bin’. A total positive outgroup attribution score consisted of the total number of positive descriptors to the Black and Middle‐Eastern children separately and combined (potential range 0–10). Negative outgroup attribution scores were calculated separately and together for the Black and Middle Eastern target children and consisted of the total number of negative descriptors assigned to these children (potential range 0–10).

### Analyses

Preliminary analyses were conducted to check the distribution of the variables. Outliers (*z*‐score > |3.29|) were found for maternal color‐conscious practices (two outliers for observed, none for self‐reported), and for child outgroup preference (one outlier). These outliers were winsorized to bring them closer to the rest of the score distribution (Tabachnick & Fidell, 1996). In addition, descriptive statistics of observed color‐conscious practices in response to various picture types were calculated and tested for differences, using repeated‐measures ANOVAs and paired‐sample t‐tests. To test the two hypotheses regarding the association of maternal color‐conscious practices and attitudes with more positive (H1) and less negative child interethnic attitudes (H2), we calculated Pearson correlations between the maternal and child variables, followed up with regression analyses in case of multiple significant correlations with one outcome variable. In addition, we conducted a MANOVA to test the potential interactive effects of different maternal color‐consciousness measures in relation to the four child outcomes (H3).

Finally we used *K*‐means cluster analysis to test the hypothesis that higher maternal color‐conscious practices and attitudes, more positive and less negative child interethnic attitudes would cluster within individual families (H4). The NbClust package in R was used to estimate the recommended number of clusters according to 30 popular indices (Charrad et al., 2014). Euclidean distance was used as the dissimilarity measure. The *K*‐means cluster analysis was performed on the standardized variables using SPSS’s default Lloyd algorithm.

## RESULTS

Table 1 shows the descriptive statistics for the picture book variables. Preliminary analyses of the observed color‐conscious practices mothers showed during book reading, revealed that mothers made on average between one and two statements reflecting color‐consciousness during book reading (noting that per theme within pictures, multiple references to race or ethnicity were only counted once). In addition, we found that 28% of mothers made no such statements at all, 63% made no color‐conscious statements about the Black characters, and 43% made no color‐conscious statements about the Middle‐Eastern characters.

**TABLE 1:** Descriptive statistics of picture book color‐conscious practices: Numbers of statements^a^ for separate book sections

Variable	Range^b^	*M* (*SD*)^b^
Total number of color‐conscious statements (0 = 28%)	0–13	1.52 (1.76)
About Black characters (0 = 63%)	0–5	0.51 (0.79)
About Middle Eastern characters (0 = 43%)	0–8	1.01 (1.18)
Picture 1 (all children in a row, no context)	0–4	0.98 (1.22)
About Black characters	0–2	0.38 (0.58)
About Middle Eastern characters	0–3	0.61 (0.76)
Pictures 2–7 (single children in ambiguous situations)	0–6	0.14 (0.59)
About Black characters	0–3	0.06 (0.31)
About Middle Eastern characters	0–3	0.08 (0.36)
Pictures 8–10 (all children in cultural context)	0–4	0.48 (0.81)
About Black characters	0–2	0.17 (0.39)
About Middle Eastern characters	0–3	0.31 (0.59)

^a^ Note that the absence/presence of each category of statements was counted per picture, so that multiple statements within one category in relation to the same picture were only counted once (see also Method section).

^b^ Statistics obtained before winsorizing (actual observed range).

Because the picture book is a new instrument, we also explored the number of statements made in response to the three picture types: the first picture that shows all the six children standing next to each other, pictures 2 to 7 that show single children in ambiguous situations, and pictures 8 to 10 that show all children in explicitly cultural settings.

Repeated‐measures ANOVAs showed that picture type was related to the number of color‐conscious comments mothers made (combining Black and Middle Eastern characters), *F*(2, 136) = 35.89, *p* < .000, $\eta_{p}^{2}$ = .35, and that all within‐subjects contrasts were significant, with mothers making most color‐conscious statements in response to the first picture with all children without context, followed by the cultural pictures with all children, followed by the single‐child pictures (all contrasts *p* < .01). We also compared the three cultural pictures, and again found a significant effect of picture on color‐conscious statements, *F*(2, 136) = 6.41, *p* < .001, $\eta_{p}^{2}$ = .09, with mothers making more such statements in response to the picture showing the Middle‐Eastern Dutch cultural scene than in response to the White Dutch or Afro‐Dutch cultural scenes (both contrasts *p* < .05).

Paired‐sample *t*‐tests showed that mothers made significantly more color‐conscious statements about the Middle‐Eastern characters than about the Black characters in the book, *t*(137) = −6.41, *p* < .01. This was also the case when looking at color‐conscious statements in response to the first group picture without context, and for the cultural pictures (both *p* < .01), but not for the single‐child pictures (*p* = .44).

Table 2 shows the correlations between the main study variables. Looking at the correlations between maternal variables, only the two self‐reported measures were significantly correlated. Most of the child outcome variables were significantly intercorrelated in the expected directions.

**TABLE 2:** Correlations among study variables

Variable	1	2	3	4	5	6
1. MCC Practices Observation	—					
2. MCC Practices Questionnaire	−.01	—				
3. MCC Attitudes Questionnaire	.09	.21*	—			
4. C Outgroup preference	−.06	.10	.13	—		
5. C Outgroup rejection	.02	−.07	−.05	−.02	—	
6. C Positive Outgroup Attitudes	−.02	.10	.15	.28**	−.09	—
7. C Negative Outgroup Attitudes	−.17*	−.20*	−.10	−.23**	.24**	−.38**

Abbreviations: C, child; MCC, maternal color‐consciousness.

* *p* < .05

** *p* < .01.

Hypotheses 1 and 2, reflecting the expectation that maternal color‐consciousness practices and attitudes to be significantly correlated with child interethnic attitude measures, were only partly confirmed, with both maternal observed and self‐reported color‐conscious practices significantly related to less negative outgroup attitudes in children.

We then examined child gender, child age, family income, maternal education, and ethnic‐racial diversity in the child's school in relation to the study variables to decide which ones should be included in the multivariate analyses. Out of 35 associations tested, only six were significant. Girls scored higher than boys on outgroup preference and positive attitudes toward the outgroups (*p* < .01), and children in more ethnically diverse schools had more negative outgroup attitudes (*p* < .05), and had mothers who showed lower observed color‐consciousness (*p* < .01). In addition, more highly educated mothers scored higher on self‐reported color‐conscious attitudes (*p* < .05), and mothers of older children scored higher on self‐reported color‐conscious practices (*p* < .05). To better understand the impact of these potential covariates on our findings in further analyses, we conducted three versions of each of the multivariate analyses with predictor and outcome variables: one without covariates, one with only school diversity as covariate (as the only variable related to both a predictor and an outcome variable), and one with all covariates significantly related to one or more study variables (i.e., child gender, child age, maternal education, and school diversity).

Two linear regression analyses were performed to test whether the significant bivariate associations would hold after correcting for the identified covariates. The association of child negative outgroup attitudes with the two maternal color‐consciousness variables dropped just below significance (*p* value .056 for observed color‐consciousness, and .064 for self‐reported color‐consciousness).

The maternal color‐consciousness variables regarding the Black and Middle Eastern characters in the picture book separately were significantly interrelated (*r* = .60, *p* < .01). Matching the maternal color‐consciousness outgroup with the child attitudes outgroups, we found the same pattern as for the overall outgroup analyses, but only for the Black outgroup: maternal Black color‐consciousness was significantly related to less negative Black outgroup attitudes in children (*r* = −.21, *p* < .05). Maternal Middle‐Eastern color‐consciousness was not related to any of the child measures regarding the Middle‐Eastern outgroup.

Hypothesis 3 regarding the potential interactive effects of the three different measures of maternal color‐consciousness on the four child outgroup attitude variables was tested with a MANOVA with a full‐factorial model. This analysis revealed no main effects of the separate maternal variables, nor any significant interaction effect between the different color‐consciousness measures: self‐reported attitudes‐by‐self‐reported practices, *F*(4, 127) = 0.69, *p* = .60, $\eta_{p}^{2}$ = .02, self‐reported attitudes‐by‐observed practices, *F*(4, 127) = 0.33, *p* = .86, $\eta_{p}^{2}$ = .01, self‐reported practices‐by‐observed practices, *F*(4, 127) = 0.83, *p* = .51, $\eta_{p}^{2}$ = .03, and the three‐way interaction, *F*(4, 127) = 1.34, *p* = .26, $\eta_{p}^{2}$ = .04. The inclusion of covariates did not change these results.

Finally, we tested Hypothesis 4 regarding the clustering of the main variables with *K*‐means cluster analysis. The NbClust package in R showed that a two‐cluster solution was supported by eight indices, a three‐cluster solution was supported by four indices, and a four‐cluster solution was supported by five indices, as was a 15‐cluster solution. All other solutions were supported by only one index. Given these results, and applying the parsimony principle, we report the two‐cluster solution (Table 3).

**TABLE 3:** Results of *K*‐means cluster analysis

Variable	Cluster 1 (*N* = 74)	Cluster 2 (*N* = 64)	*F*‐test^a^
Maternal color‐conscious practices—O	−.42	.37	25.41**
Maternal color‐conscious practices—Q	−.44	.38	26.98**
Maternal color‐conscious attitudes	−.25	.21	7.64**
Child outgroup preference	−.40	.35	21.98**
Child outgroup rejection	.07	−.06	0.56
Child positive outgroup attitudes	−.62	.54	68.21**
Child negative outgroup attitudes	.65	−.56	79.26**

Note

Numbers represent *z* values for cluster centers.

^a^ Significance of the variable's contribution to the clustering.

** *p* < .01.

The first cluster (*N* = 74) reflects families with mothers higher on color‐consciousness practices and attitudes, and with children higher on outgroup preference and positivity, and lower on outgroup negativity (but not rejection). The second cluster (*N* = 64) reflects families with the exact opposite pattern. All variables except for child outgroup rejection distinguished significantly between the two clusters. Cluster analysis with outgroup‐specific variables revealed the same pattern for the Black and the Middle‐Eastern outgroup, with two clusters distinguishing between a group of families characterized by high color‐consciousness and more positive/less negative child outgroup scores, and a group of families with the opposite characteristics.

All results were the same (in terms of significance levels) when using the total score for self‐reported maternal color‐conscious practices, except that it did not correlate significantly with self‐reported maternal color‐conscious attitudes.

## DISCUSSION

The current study is the first to examine maternal color‐conscious practices and attitudes in White Dutch families in the Netherlands, with the goal to address maternal openness about ethnic‐racial (power) differences as key prerequisites for anti‐racist parenting. The results showed significant associations of color‐conscious practices and attitudes with child attitudes toward ethnic‐racial outgroups, but not consistently across measures and analyses. Using a variable‐centered approach, maternal color‐conscious practices (but not attitudes) were associated with less negative attitudes (but not more positive ones) toward ethnic‐racial outgroups in children. Using a person‐centered approach, maternal color‐conscious practices and attitudes clustered with more positive and less negative child attitudes toward ethnic‐racial outgroups in the expected constellations.

The first hypothesis regarding the bivariate association between maternal color‐conscious practices and attitudes on the one hand and more positive child attitudes about ethnic‐racial outgroups on the other hand was not supported. None of the correlations relevant to this hypothesis were significant. The second hypothesis about maternal color‐consciousness in relation to less negative child attitudes was partially supported: both observed and self‐reported maternal color‐conscious practices (but not attitudes) were related to lower levels of child negative attitudes toward ethnic‐racial outgroups (but not outgroup rejection). Apparently, links between maternal ethnic‐racial socialization dimensions and children's attitudes about ethnic‐racial outgroups may vary depending on the specific types of construct and assessment. Although the use of three measures to assess different aspects of maternal color‐consciousness is a strength of the current study, the low or absent associations between the measures raise questions about the coherence of the color‐consciousness construct. On the other hand, weak associations between observed and self‐reported behaviors, and between attitudes and behaviors are very common in psychological research (e.g., Hendriks et al., 2018; Sheeran, 2002) and may also reflect actually distinct aspects of color‐consciousness elicited by different ways of probing their presence. Because this construct is rather new in empirical research on parenting, especially in White families, the development of a coherent set of measures reflecting well‐defined and clearly delineated aspects of color‐consciousness would be very helpful to the field. This set should include a clear distinction between color‐evasive, power‐evasive, and anti‐racist socialization. We also want to note though that the cluster analysis did reveal meaningful patterns of co‐occurring levels of the different measures, showing the added value of conducting person‐oriented analyses in addition to variable‐oriented analyses.

The third hypothesis regarding potential interactive effects of different types of color‐conscious practices and attitudes was not confirmed. Thus, the effect of one type of maternal color‐consciousness was not amplified by the presence of another type. It may be that the presence of any form of parental color‐consciousness is what is important in reducing child prejudice, and that it is not necessary that this parenting characteristic is displayed in various ways to be effective. This suggest that as long as the key message of color‐consciousness is present in parent–child interactions, variations in the type and number of ways in which it is present are not relevant. However, to more fully understand which ethnic‐racial socialization elements predict which child outcomes in which combinations, the development and testing of more symmetrical measures of behaviors and attitudes (i.e., with aligned and distinct content regarding different dimensions) would be useful. Including more targeted measures of explicitly anti‐racist socialization practices would also be recommended. The recent work by Gillen‐O’Neel et al. (2021) provides some interesting categories of ethnic‐racial socialization that could be useful for future studies.

The fourth hypothesis about clustering of color‐conscious socialization with more positive and less negative child ethnic‐racial attitudes was supported. Our person‐centered approach to the data confirmed the notion of a coherent set of color‐conscious socialization dimensions, as it revealed two subpopulations reflecting a group of families in which mothers show color‐conscious practices and attitudes, with children who show more positive and less negative interethnic attitudes, and a group of families in which this pattern is reversed. The identification of these two groups is consistent with the notion of color‐conscious socialization co‐occurring with low prejudice in families as found in other studies with variable‐centered approaches (e.g., Pahlke et al., 2012; Perry et al., 2019; Vittrup & Holden, 2011). Finding out more about these distinct groups in terms of their specific ethnic‐racial socialization attitudes and practices is crucial to understanding how which parents contribute to less prejudiced ethnic‐racial attitudes in their children, and how this can inform anti‐racist family interventions.

Reflecting on the differences between the results from the variable‐centered analyses (correlation, regression) and person‐centered analyses (clustering), we note that the latter leaves room for non‐linear patterns of association of rank orders, whereas the former does not. It seems that the associations between parent and child characteristics tested here do not all follow linear patterns in which higher scores on one variable predict higher scores on another variable. Instead, we identified two groups of families with specific patterns of characteristics (lower on some, higher on others, but not necessarily with corresponding rank orders of the distributions), that correspond with the hypothesized patterns of association. In addition, whereas variable‐centered analysis assume that a study sample reflects one population in which patterns of associations are the same across the board, person‐centered analyses does not. The latter identifies specific subgroups (clusters) within the sample who show their own specific patterns of characteristics. Thus, our results show that more color‐conscious parenting does not uniformly predict lower child prejudice, but instead specific levels of these parent and child characteristics are found to coincide in two distinct groups of families.

The current study also showed that several sociodemographic characteristics of families are relevant for understanding ethnic‐racial socialization and attitudes. Girls were found to have more positive attitudes toward ethnic‐racial outgroups, which is consistent with previous findings (e.g., Verkuyten & Thijs, 2001), and likely reflects girls’ stronger general prosocial tendencies. In addition mothers with higher educational levels reported less power‐evasive attitudes, which has also been found previously by Hagerman (2014). Furthermore, ethnic‐racial school diversity was related to less observed maternal color‐conscious practices and more negative child attitudes toward outgroups. This finding seems to be in contrast with insights and empirical evidence regarding intergroup contact theory that generally show that contact with outgroups is beneficial for children's attitudes toward those groups (Pettigrew & Tropp, 2006). However, our variable does not cover contact per se (just presence of diversity), and also does not address whether the key conditions for positive intergroup contact are met. Thus, there is a possibility that actual intergroup contact was low even in children in ethnic‐racially diverse schools, or that intergroup contact was actually negative and, therefore, did not predict more positive attitudes. In addition, it has been argued that school diversity in and of itself is not enough to reduce prejudice in children but that the ways that schools address and support inclusivity are more important (Thijs & Verkuyten, 2014). There is also evidence that the nature of ethnic diversity in terms of numbers of ethnic groups and numbers of children from specific groups need to be specified to understand the effect. A few larger ethnic groups can exacerbate ingroup‐outgroup distinctions, whereas smaller groups might dilute this effect (e.g., Moody, 2001). Unfortunately, our assessment of school diversity did not cover such details. In the current study this restricted variable was only included as a covariate and not part of our main hypotheses, so that strong inferences cannot be made about the role of school diversity or intergroup contact on the main concepts of interest.

The new picture book used in the present study proved to be useful in uncovering aspects of parental ethnic‐racial socialization that are meaningfully related to children's ethnic‐racial attitudes. Using this measure, the Dutch sample showed less widespread color‐evasive practices than is usually reported in US samples (Pahlke et al., 2012; Vittrup & Holden, 2011), which allowed for more informative analyses of relations with child outcomes.

This may also mean that anti‐racist parenting interventions that were unsuccessful in US families due to parents’ unwillingness to give up their color‐evasive socialization approach might actually be more fruitful in the Netherlands, and potentially other European countries. The picture book used in the present study could be used to guide parents in how they can discuss ethnic‐racial topics with their children. However, we first need more detailed information about (Dutch) parents’ responses to the book to fully understand what it elicits in which families. Further analyses of the mother‐child conversations need to systematically examine the precise content of the statements in terms of which ethnic‐racial characteristic is mentioned, the valence of the statements (positive, neutral or negative), and references to (anti)racism.

Notably, mothers were much more likely to mention ethnic‐racial characteristics of the Middle‐Eastern characters than of the Black characters. This could reflect a more fundamental unease felt by White parents with regard to addressing a skin color markedly different from their own, which in turn may reflect a particular fear of being perceived as racist, whereas identifying Middle‐Eastern people based on their appearance is far less controversial and less associated with the term racism (in the Dutch context). Interestingly, this clear difference in frequencies of statements did not translate into different patterns of findings for the Black and the Middle‐Eastern outgroups. It appears that although the average levels of color‐consciousness varies between different ethnic‐racial outgroups, the associations and clustering with children's ethnic‐racial outgroup attitudes for these specific groups are mostly similar.

The current study has several strengths, including the use of observational measures for maternal color‐conscious practices, and the investigation of maternal practices as well as attitudes in relation to child attitudes toward ethnic‐racial outgroups. Another strength is that the study extended ethnic‐racial socialization research to the Netherlands. The study also has some limitations. The sample consisted of highly educated mothers who were motivated to participate in a study on ethnic diversity in Dutch society, and were, therefore, not representative of the Dutch population. Given that socioeconomic status and ethnic‐racial attitudes are related (e.g., Hagerman, 2014), more diverse samples in terms of education and income are needed to understand the full range of racial socialization practices and their relation with child prejudice. In addition, a larger sample might have been better able to detect smaller effect sizes, and have more statistical power in analyses with multiple covariates. Other limitations include the cross‐sectional nature of the data that include only information on mothers, and not fathers, teachers, or peers, as well as the focus on the majority group's perspective rather than the outgroups’ perspectives. Finally, although the picture book used in this study represents an innovative and promising approach to studying maternal color‐conscious messages to children, more detailed analyses of the content of mothers’ messages would provide more in‐depth information about the intergenerational transmission of attitudes about ethnic‐racial outgroups.

In conclusion, the notion of color‐conscious socialization appears to be a worthwhile concept to explore further in families from a society's dominant ethnic group in the quest for understanding the basis for parenting practices that contribute to a new generation of color‐conscious and anti‐racist individuals. However, for a more contextualized understanding of these processes, we may need to develop new terminology that does justice to phenomena of ethnic‐racial marginalization that go beyond issues of skin color, and that are common in countries like the Netherlands. To continue on this quest, we need scholars who are motivated to invest in a wide variety of study designs, populations, measures and analyses and delve deep into all the mechanisms that are at play in the family context. A strong empirical base with room for quantitative and qualitative approaches from all over the world will foster increasingly strong theories, which in turn will foster more sophisticated research. We look forward to seeing the current surge in interest in this field materialize into a globally inclusive research tradition that will answer the many questions that society is waiting for developmental scientists to answer.
